# Development and Measurement of the Treatment Perceptions Survey (TPS) for Clients with Substance Use Disorders

**DOI:** 10.1007/s11414-021-09776-y

**Published:** 2022-02-25

**Authors:** Cheryl Teruya, Vandana Joshi, Darren Urada, Tom Trabin, Isabel Iturrios-Fourzan, Yu-Chuang Huang

**Affiliations:** 1 University of California, 10911 Weyburn Ave., Suite 200, CA 90024 Los Angeles, USA; 2 Behavioral Health Concepts/California EQRO, 5901 Christie Ave., Suite 502, Emeryville, CA 94608 USA

## Abstract

Statistical reliability of the Treatment Perceptions Survey (TPS) questionnaire was examined using data from 19 California counties. The 14-item TPS was designed for clients receiving substance use disorder services at publicly funded community-based programs. The TPS is being used for evaluation of the State’s 1115 Medicaid Waiver, external quality review of county-based systems of care, and quality improvement efforts. The survey addresses four domains of access to care, quality of care, care coordination, and general satisfaction that each include multiple items, plus a single item focused on self-reported outcome. Reliability test results of the four domains as composite measures were statistically significant. General satisfaction ratings were the best predictor of self-reported outcome in a path analysis model, followed by ratings of care coordination and quality of care. Separate analyses of TPS data from clients receiving specialty mental health services suggest the questionnaire can also be used reliably in mental health settings.

## Introduction

In 2015, the Centers for Medicare and Medicaid Services (CMS) approved the request from the California Department of Health Care Services (DHCS) for a 5-year Sect. 1115 Waiver to initiate an innovative demonstration program called the Drug Medi-Cal^1^ Organized Delivery System (DMC-ODS). The waiver was designed to transform publicly funded specialty substance use disorder (SUD) treatment delivery systems in the state. Measuring client perceptions of and satisfaction with SUD treatment services is an integral part of the evaluation of the waiver, quality improvement efforts, and provision of client-centered care.

Currently, a client perceptions of care survey is not required for grantees of state Substance Abuse Prevention and Treatment Block Grant funds as it is for grantees of Community Mental Health Services Block Grant funds.^1–4^ In addition, a standard survey in widespread use for clients receiving specialty SUD services does not exist as it does for clients receiving specialty mental health (MH) services (e.g., Mental Health Statistics Improvement Program [MHSIP] Consumer Survey^2^). In contrast, the MHSIP survey has been used extensively across the nation for several decades, and is used by states, including California, to collect data for reporting on the federally determined National Outcome Measures in order to receive federal MHBG funding. Multiple versions of the MHSIP survey are currently in use across the country with clients receiving specialty MH services. They have also been used, albeit to a much lesser extent, with clients receiving SUD treatment services.^5^ Some of the items have also been used in several California counties (e.g., San Francisco, Contra Costa, Solano) with populations receiving specialty SUD treatment.^5^

The 36-item MHSIP Consumer Survey used in California with clients receiving specialty MH includes seven domains: General Satisfaction, Perception of Access, Perception of Quality and Appropriateness, Perception of Participation in Treatment Planning, Perception of Outcomes of Services, Perception of Functioning, and Perceptions of Social Connectedness. These 36 items have been tested for their psychometric properties and have yielded statistically reliable results for three factors and seven domain scales.^1,2^ At the same time, clients may find that the 36-item survey items is too long and burdensome to complete.^6^ Many stakeholders and clients have expressed concern about the burden of completing lengthy surveys.^7^

Some surveys have been developed for use with clients receiving behavioral health (BH) services more generally. For example, the Modular Survey (also known as the Perception of Care survey^8,9^), the Consumer Assessment of Healthcare Providers and Systems and the Consumer Assessment of Behavioral Health Survey^10^ were designed for use in both MH and SUD treatment settings. Meanwhile, the Experience of Care and Health Outcomes survey was developed to collect client experiences with BH care and services,^11,12^ provided through managed care organizations or managed BH care organizations. However, these surveys do not meet many of the specifications requested by California county SUD administrators and other stakeholders, who communicated to the survey developers during public meetings that they wanted a one-page user-friendly tool to collect meaningful and actionable information from the client perspective. To the authors’ knowledge, a validated client perception of care survey developed specifically for uniform statewide use in publicly funded specialty SUD treatment provided in community-based settings is not available.^11^

The client Treatment Perceptions Survey (TPS) was developed to fill this gap for the SUD treatment field. It comes at a time when publicly funded SUD treatment and systems of care are becoming less program-driven and more client-centered. Consequently, client perceptions of their SUD treatment are becoming more valued considerations in how that treatment is delivered and evaluated. This shift is explicitly embodied in the Special Terms and Conditions of the DMC-ODS Waiver.

The TPS is a shorter questionnaire than the MHSIP survey and consists of only 14 client-rated items. The items are grouped into four domains that are included in the MHSIP survey, and a single perception of outcome item. Seven of these items were adapted from the MHSIP survey, and the remaining seven were developed for the DMC-ODS Waiver evaluation or taken from San Francisco County’s satisfaction survey for clients receiving behavioral health services. The TPS was designed for clients receiving SUD services. The questionnaire developers solicited and incorporated input from DHCS and other stakeholders (e.g., county administrators) into the survey. An early version of the questionnaire was pilot tested in Marin County prior to the county’s implementation of the waiver to collect baseline data. Feedback received from county administrators and providers indicated that all clients who were offered the questionnaire were willing to complete it, and many were “quite enthusiastic to have their opinions solicited” (Catherine Condon, MPH, email communication, April 6, 2017); no modifications to the content of the survey questionnaire were recommended.

The purpose of the present study is to examine the measurement properties of the TPS questionnaire developed from a client-centered perspective specifically for adult clients receiving publicly funded specialty treatment for SUD under the DMC-ODS Waiver. DHCS requires each county implementing the DMC-ODS Waiver to administer the TPS to both adults (age 18 and older) and youth (e.g., ages 12–18) at least once annually during a specified 5-day period if they had been providing DMC-ODS services for at least 1 month prior to the survey period. In calendar year (CY) 2017, the first year of the TPS, seven counties administered the surveys and returned questionnaires or submitted data for 9027 adults to UCLA for data analysis. In CY2018, the first year the TPS questionnaire for youth was used, 19 counties participated in the TPS and returned questionnaires or submitted data for 15,259 adults and 669 youth.

Given the requirement that the DMC-ODS Waiver county use the TPS in SUD treatment settings and the emerging interest in its use in MH settings (particularly due to its shorter length compared to the MHSIP), the authors deemed it important to analyze and report the measurement properties of this tool. Some of the TPS items have been adapted from the widely used MHSIP survey, which has established psychometric properties for self-reported satisfaction in six or seven domains.^2,13^ However, the measurement properties of the 14 TPS items that are grouped into four domains, namely, Access to Care, Quality of Care, Care Coordination, and General Satisfaction, have not yet been examined statistically. Therefore, one goal of the present study is to test the statistical reliability and validity of the TPS items across demographic categories such as age group, race/ethnicity, gender identity, and primary language groups within SUD treatment settings. In addition, some counties such as Monterey also used the TPS in selected MH settings with minor modifications in wording. The statistical reliability of the TPS for clients receiving MH services in Monterey County is also reported here.

## Methods

### Survey instrument and procedures

The TPS for adults was developed based on a one-page Treatment Satisfaction Survey created by San Francisco County, which included seven items adapted from the MHSIP Consumer Survey. Input from DHCS and other groups of stakeholders (e.g., county BH administrators) were incorporated into the questionnaire and survey administration procedures. The TPS was designed to serve multiple purposes: (1) fulfill a county’s External Quality Review requirement related to conducting a client perception of care/satisfaction survey at least annually using a validated tool, (2) address the client perception of care component of the evaluation required by the CMS required evaluation of the DMC-ODS demonstration, and (3) support DMC-ODS quality improvement efforts and provide key information on the impacts of the new continuum of care. The TPS was also designed to be user-friendly and to collect meaningful, actionable data using an administration process that minimizes the burden on counties/providers as well as clients.

As shown in Table 1, the survey includes 14 items addressing client perception of Access to Care (two items), Quality of Care (five items), Outcome (one item), Care Coordination (two items), and General Satisfaction (four items).

**Table 1:** TPS domains and survey items

TPS domains	Survey items
Access	1. The location was convenient (public transportation, distance, parking, etc.)
Access	2. Services were available when I needed them
Quality	3. I chose the treatment goals with my provider’s help
Quality	4. Staff gave me enough time in my treatment sessions
Quality	5. Staff treated me with respect
Quality	6. Staff spoke to me in a way I understood
Quality	7. Staff were sensitive to my cultural background (race/ethnicity, religion, language, etc.)
Care coordination	8. Staff here work with my physical health care providers to support my wellness
Care coordination	9. Staff here work with my mental health care providers to support my wellness
Outcome	10. As a direct result of the services I am receiving, I am better able to do things that I want to do
General satisfaction	11. I felt welcomed here
General satisfaction	12. Overall, I am satisfied with the services I received
General satisfaction	13. I was able to get all the help/services that I needed
General satisfaction	14. I would recommend this agency to a friend or family member

Survey responses indicate the extent to which respondents agree or disagree with the statements using a five-point Likert scale (from strongly agree to strongly disagree) or whether the respondents consider the statement to be “not applicable.” The survey also collects demographic/background information (e.g., gender identity, age group, race/ethnicity, length of time receiving services at the treatment program). In addition, there is a section where respondents may write their comments. TPS questionnaires are available in 13 languages (English, Spanish, Chinese, Tagalog, Farsi, Arabic, Russian, Hmong, Korean, Eastern Armenian, Western Armenian, Vietnamese, and Cambodian) and in one-page and larger font versions. Counties or provider staff fill in the treatment provider identification number assigned by DHCS and treatment setting (outpatient/intensive outpatient, residential, opioid/narcotic treatment program [OTP/NTP], detox/withdrawal management [standalone], partial hospitalization) prior to offering the questionnaires to clients to complete. Participation in the TPS is strictly voluntary and anonymous. A youth version of the TPS questionnaire is also available.

### Data collection and summary reports

Counties participating in the DMC-ODS Waiver are required to have their network of treatment provider survey clients at least annually during a 5-day period specified by DHCS. The questionnaires and other TPS-related information and resources are posted on the TPS website.^3^ Administrators at the county level are responsible for providing local instructions, coordinating the TPS in their respective counties, and submitting the client responses, either as paper questionnaires or data files, to the DMC-ODS Waiver evaluation team for scanning (if needed) and analysis. The waiver evaluation team prepares county- and program-level summary reports and statewide reports for DHCS and the external quality review organization.

### Sample

The present analysis used data from the second TPS administration to adult clients in 19 counties in CY 2018.^4^ All providers in the counties’ SUD treatment provider network (county-operated and/or county-contracted) were instructed to offer the survey questionnaire to every client who presented in person to receive substance use services during the designated survey period of October 1–5, 2018; this group of clients comprise the convenience sample. Clients were asked to complete only one survey questionnaire even if they received services more than once during the survey period (e.g., clients in residential treatment). If a client received services in more than one treatment setting (e.g., residential and opioid/narcotic treatment program) during the five-day survey period, they were offered a survey questionnaire in each treatment setting.

### Analysis

Cronbach’s alpha was tested on 13 out of the 14 survey items to measure internal consistency of TPS domain scales across various demographic categories (e.g., age group, gender identity, race/ethnicity, language of the questionnaire). These 13 items were hypothesized to measure four TPS domains (Access to Care, Quality of Care, Care Coordination, and General Satisfaction). The fourteenth question was a single item that was hypothesized to measure clients’ perceptions of “Outcome” as a result of receiving treatment services, and therefore was not tested for its psychometric properties. Cronbach’s alpha indicates the extent to which a set of items are closely inter-related as a group or scale, with a score ranging between zero and one. A value equal to or greater than 0.70 is considered a reliable scale.^14,15^.

However, since a high Cronbach’s alpha does not necessarily imply that the underlying measure is multdimensional, exploratory factor analysis (EFA) was subsequently conducted on the 13 items to assess for multidimensionality. Factor analysis explores factors from a group of variables with high inter-correlations. Exploratory factor analysis provides factor loadings, which are the correlation of the original variables with the factor. Factor loadings help determine the relative importance of a particular variable to a factor.

Next, confirmatory factor analysis (CFA) was conducted to test the statistical validity of the EFA model. The validity of a CFA model is displayed by parsimonious fit model indices, including adjusted goodness of fit index (AGFI), confirmatory fit index (CFI), and root-mean-square error of approximation (RMSEA).

The AGFI was calculated to examine the proportion of variance accounted for by the estimated population covariance^16^ and how closely the model comes to replicating the observed covariance matrix.^15^ The values for AGFI range between 0.0 and 1.0, and values of 0.90 or greater indicate well-fitting models.

The CFI index was calculated because it is one of the fit indices that is least affected by small sample sizes.^16,17^ This statistic assumes that all latent variables are uncorrelated (null/independence model) and compares the sample covariance matrix with this null model. Values for CFI also range between 0.0 and 1.0, with values closer to 1.0 indicating good fit. A value of CFI equal to or greater than 0.95 is presently recognized as indicative of good fit.^18^ Given the impact of sample size on the AGFI fit index, it was not relied upon as a stand-alone index, but the AGFI fit index is reported in covariance structure analyses and analyzed in conjunction with other fit indices, such as the CFI, reported in the model.

The RMSEA indicates how well the model with unknown but optimally chosen parameter estimates would fit the population covariance matrix.^19^ It is regarded as one of the most informative fit indices^20^ due to its sensitivity to the number of estimated parameters in the model. An RMSEA in the range of 0.05 to 0.10 is considered to indicate a good fit, and values above 0.10 indicate a poor fit.^21^

Structural equation modeling (SEM) path analysis was conducted to predict the Outcome variable by the four TPS domain scales, controlling for background factors and variables.

Monterey County used the TPS among clients receiving MH services in CY2018.^5^ Analysis was conducted to examine the internal consistency for the same 13 out of the 14 TPS items addressing the four domains among adults (*N* = 147). Due to the small sample size, factor analyses could not be conducted on this sub-set as any reliable factor analyses require data from at least 300 clients.

## Results

### Survey response rate and respondent characteristics

As shown in Table 2, a total of 15,259 adult survey questionnaires were received from 19 counties. The response rate was conservatively estimated at 41.0%.^6^ The sample was comprised of 60% males and 40% females. The highest percentage of survey questionnaires were received from respondents between the ages of 26 and 35, and nearly three quarters were either White or Latinx. Approximately 97% of the survey questionnaires received were in English and 3% in Spanish, although questionnaires were available in 13 languages.

**Table 2:** Demographic distribution of survey respondents

Demographics	*N*	%
Age group		
18–25	1,368	9.6%
26–35	4,365	30.6%
36–45	3,334	23.4%
46–55	2,743	19.3%
56 +	2,433	17.1%
Total	14,243	100.0%
Gender identity		
Male	8930	59.7%
Female	5896	39.4%
Other	122	0.8%
Total	14,948	100.0%
Race/Ethnicity		
American Indian/Alaska Native	353	2.4%
Asian	233	1.6%
Black/African American	2105	14.1%
Latinx	4690	31.5%
Native Hawaiian/Pacific Islander	117	0.8%
White	6401	43.0%
Other	990	6.6%
Total	14,889	100.0%
Survey questionnaire language		
English	15,224	96.6%
Spanish	535	3.4%
Total	15,759	100.0%

### Reliability of TPS domain scales

Three out of the four TPS domain scales had a Cronbach’s alpha greater than 0.70 as shown in Table 3. The Access to Care domain had an alpha value of 0.62, which is acceptable but not optimal. One of the reasons for the lower alpha value of this scale could be because in geographically large counties with a mixture of urban and rural areas, the DMC-ODS might offer timely first appointments for all prospective clients but the location may not be convenient for some of them.

**Table 3:** Internal consistency (Cronbach’s alpha) of TPS items by domain

Domains	Number of Items	Alpha
Access	2	0.62
Quality	5	0.89
Care coordination	2	0.89
General satisfaction	4	0.91

Table 4 shows the alpha value of the four TPS domain scales by various demographic categories. The Cronbach’s alpha values for three out of the four scales — Quality of Care, Care Coordination, and General Satisfaction — were consistently high (greater than 0.70) across all categories of age group, gender identity, race/ethnicity, and language of the survey questionnaire. Cronbach’s alpha for the Access to Care scale across the various demographic categories was between 0.57 and 0.76.

**Table 4:** Internal consistency (Cronbach’s Alpha) for TPS domains by demographic categories

Demographic categories	Access	Quality	Care coordination	General satisfaction
Age group				
18–25	0.61	0.87	0.87	0.91
26–35	0.57	0.87	0.89	0.91
36–45	0.67	0.89	0.89	0.92
46–55	0.62	0.88	0.88	0.90
56 +	0.66	0.90	0.87	0.92
Gender Identity				
Male	0.63	0.89	0.88	0.91
Female	0.59	0.88	0.89	0.91
Other	0.71	0.92	0.93	0.92
Race/Ethnicity				
American Indian/Alaska Native	0.67	0.90	0.91	0.92
Asian	0.66	0.89	0.89	0.92
Black/African American	0.64	0.89	0.88	0.91
Latinx	0.62	0.88	0.88	0.91
Native Hawaiian/Pacific Islander	0.76	0.93	0.92	0.90
White	0.59	0.88	0.89	0.91
Other	0.69	0.88	0.88	0.91
Survey language				
English	0.62	0.89	0.89	0.92
Spanish	0.64	0.87	0.83	0.87

### Exploratory factor analysis

The EFA met all the statistical requirements, including scale item responses being roughly normally distributed and having at least 50 observations per variable, as measured on a 5-point interval scale to ensure that the results are generalizable (see Table 5). The analysis yielded only one factor with an eigenvalue greater than 1 (8.47).

**Table 5:** Eigenvalues of reduced correlation matrix

	Eigenvalue	Difference	Proportion	Cumulative
1	8.48	7.93	0.88	0.88
2	0.54	0.19	0.06	0.93
3	0.36	0.07	0.04	0.97
4	0.29	0.22	0.03	1.00
5	0.07	0.02	0.01	1.01
6	0.05	0.04	0.01	1.01
7	0.01	0.00	0.00	1.01
8	0.01	0.01	0.00	1.02
9	0.00	0.01	0.00	1.02
10	− 0.01	0.01	0.00	1.01
11	− 0.02	0.01	0.00	1.01
12	− 0.03	0.00	0.00	1.01
13	− 0.03	0.03	0.00	1.01
14	− 0.06		− 0.01	1.00

The factor pattern for a forced four-factor solution (corresponding to the four TPS domains) further validates the present study’s finding that the model reliably yields a one-factor solution. As shown in Table 6, only the first factor has all positive loadings or correlations with the factor. The second factor shows two plausible positive correlations with the factor (scores 08 and 09). The third and fourth factors do not have any reasonably high positive correlations with the factor. This reinforces the present study’s conclusion that the best factor solution with these 13 survey items is a one-factor solution.

**Table 6:** Exploratory analysis — factor patterns of survey items

Survey items (abbreviated)	Factor1	Factor2	Factor3	Factor4
1. Convenient location	0.49719	0.04399	0.09706	0.18926
2. Convenient time	0.75868	− 0.00267	0.12234	0.22892
3. I chose my treatment goals	0.7223	0.09234	0.03095	0.27045
4. Staff gave me enough time	0.78112	− 0.0125	− 0.04352	0.20541
5. Treated with respect	0.79784	− 0.22998	− 0.23248	− 0.0193
6. Understood communication	0.82916	− 0.21087	− 0.31383	− 0.00907
7. Cultural sensitivity	0.75374	0.00398	− 0.2071	− 0.00207
8. Work with physical health providers	0.78782	0.40748	− 0.09556	− 0.11819
9. Work with mental health providers	0.76833	0.44144	− 0.06098	− 0.11262
10. Better able to do things	0.7841	0.02865	0.12895	− 0.0431
11. Felt welcomed	0.81235	− 0.168	0.02976	− 0.13141
12. Overall satisfied with services	0.87724	− 0.15961	0.21537	− 0.15903
13. Got the help I needed	0.83785	− 0.01864	0.1828	− 0.06666
14. Recommend agency	0.81917	− 0.13671	0.1552	− 0.10199

### Confirmatory factor analysis

CFA with four forced factors corresponding to the four TPS domains was conducted to determine the extent to which the four TPS domains were correlated with each other as latent variables. In addition, the CFA solution was used to test the significance of factor loadings on each factor.

The CFA solution converged and produced reliable statistics for the model. The four factors in the CFA model used the same 13 variables as in the EFA model, but this time specified as separate factors. The CFA model showed a very high correlation between the four factors, between 0.74 and 0.95, indicating that a one-factor solution as presented in the EFA model was a better fit to the data.^6^

However, CFA provides other significant statistics for data reduction that EFA does not. These additional statistics such as AGFI, CFI, and RMSEA available in CFA data modeling tell us whether the data was adequately reduced using factor analysis. While EFA tells us how many factors are possible in a data solution, CFA tells us how many factors are statistically reliable and if the data is statistically reduced based on the various fit indices. In the current analysis, the CFA solution is not necessarily used to measure four separate factors, but rather to understand adequate data reduction. The CFA analysis converged and provided satisfactory AGFI and CFI values of 0.91 and 0.97, respectively. The RMSEA value was 0.08, indicating that only 8% of the residual covariance remained in the data after fitting the CFA solution. In other words, more than 92% of the covariance in the data was explained using factor analysis.

### Path analysis predicting clients’ perceptions of outcome

After testing the covariance structure of the 13 survey items with EFA and CFA modeling, a final analysis was conducted to examine whether the four TPS domains significantly predicted respondents’ perceptions of whether they are better able to do things that they want to do as a result of the services they are receiving (Outcome variable). While the authors acknowledge that it is not ideal to use an item from the questionnaire as the dependent variable and use the rest of the items in the questionnaire as independent variables, the perception of Outcome item served as a proxy for actual outcomes at this early stage of analyses.

Given the one-factor solution explored in the EFA model and confirmed by the CFA model, the items in each of the four TPS domains were summed up as composite variables or scales to specify Access to Care, Quality of Care, Care Coordination, and General Satisfaction, and used in a path analysis regression model to predict the single-item Outcome variable. Since these variables had already been tested for their Cronbach’s alpha reliability, the analysis allowed the TPS domains to be used as four separate composite variables in a regression analysis using structural equation modeling.

**Figure 1:**
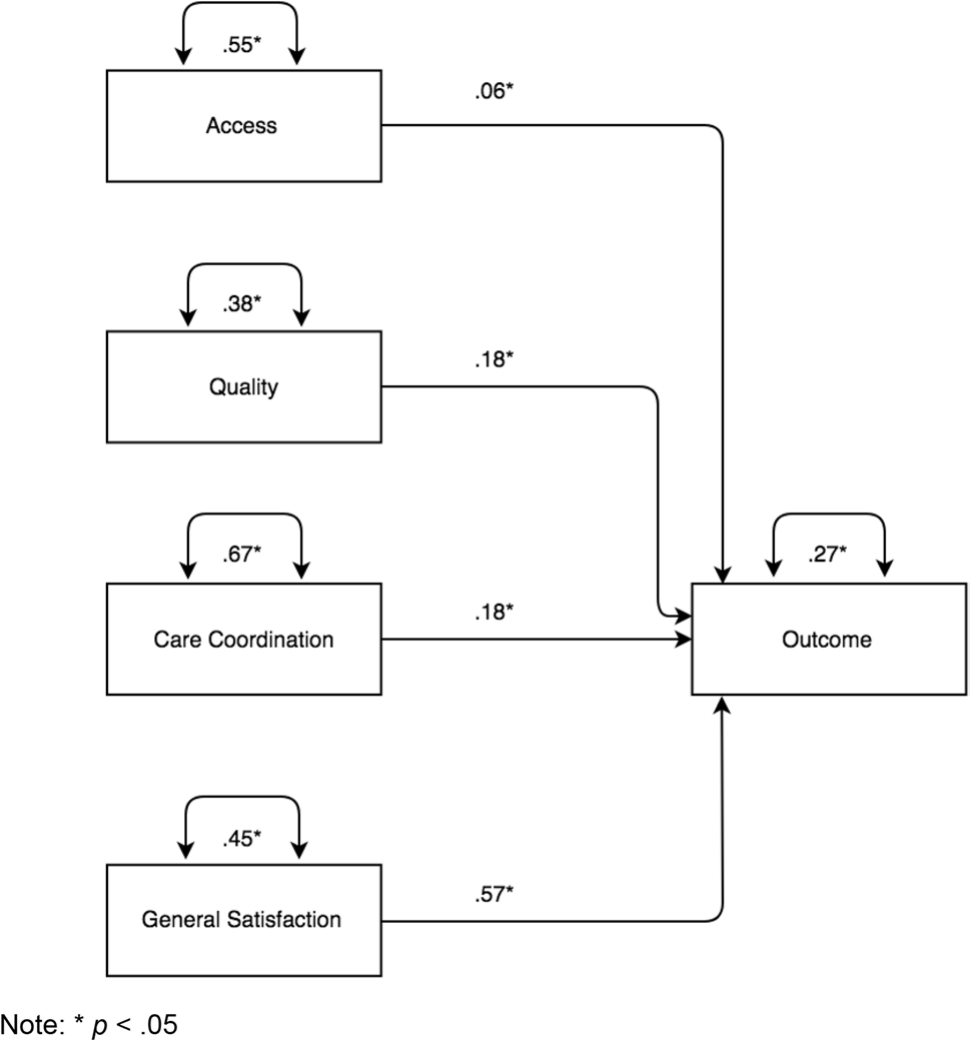
Path analysis (standardized solution). Note: *p < .05

The standardized result solution in Fig. 1 shows that all four TPS domains significantly predict the Outcome variable. This regression model was controlled for demographic variables, namely age group (reference category = 18–25-year-old), gender identity (reference category = male), race/ethnicity (reference category = White), and size of county (reference county = Los Angeles). The *R*^2^ or explained variable for this model was 59%.

The standardized regression coefficients specified in the model allowed comparison of the relative importance of each variable, adjusting for their scale of measurement. In this model, General Satisfaction was the most important predictor of Outcome with a coefficient of 0.57, followed by Quality of Care and Care Coordination, each with a coefficient of 0.18. The least important predictor of Outcome in this model was Access to Care with a coefficient of 0.06.

### Results of the TPS in mental health settings

Cronbach’s alpha was tested for the 13 items of the TPS, which had been administered to clients receiving specialty MH services in Monterey County. The results were consistent with those found among clients receiving SUD treatment services. The Cronbach’s alpha or measure of scale reliability for all four TPS domains tested were equal to or greater than 0.70 for the sample of clients receiving specialty MH services.

## Discussion

The TPS was developed for use with clients receiving SUD treatment services in publicly funded programs. Overall, the TPS results using data from 19 counties that have been participating in California’s DMC-ODS Waiver suggest the four TPS domains — Access to Care, Quality of Care, Care Coordination, and General Satisfaction — are statistically reliable and can be used as composite scales for data analysis.

Analysis of internal consistency showed congruence among the 14 survey items. The Cronbach’s alpha analysis results indicate the 13 items corresponding to four of the five TPS domains (Access to Care, Quality of Care, Care Coordination, General Satisfaction) are statistically robust and can be constructed as “measured” variables and used in regression or any other covariance analysis. While the EFA results reveal a one-factor solution, the CFA yielded substantial factor loadings and reliable fit indices. In order to improve the robustness of the current results, further replication of these analyses with different sub-populations is needed, such as clients receiving SUD services in various treatment modalities such as residential versus OTP/NTP or outpatient, or different age groups, gender identity groups, or racial/ethnic groups.

Construct validity of the proposed domain structure was not conclusively established through the EFA factor analysis because of the high inter-correlation between each of the domains as separate factors. Because there were only 13 survey items measuring four domains, it is not surprising to find a unidimensional factor for satisfaction. In addition, as alpha reliabilities held up in our analysis (with a high alpha), these domains could still be used as measured variables in a regression analysis to predict outcome. The authors acknowledge that while a shorter survey with fewer items minimizes survey respondent fatigue, it increases the risk of a unidimensional factor compared to a longer survey with many survey items to search for a multi-dimensional factor structure, which may or may not exist in several sub populations. In future studies, it may be more fruitful to test the content validity of the domains and their items. This might be done with a cognitive testing approach involving clients to determine consistent interpretation of the items’ intended meaning.

The current study provides a statistical framework for researchers to explore clients’ perceptions of Outcome in substance use and possibly MH settings. The authors acknowledge that using one item as the dependent variable and the rest of the survey items as independent variables is not ideal. These results should be interpreted with caution given that Outcome has a high correlation with the other items in this analysis. Since the TPS survey is anonymous, clients’ actual outcomes in SUD treatment such as length of stay, frequency of substance use and abstinence could not be linked to their perceptions of care. Therefore, as a proxy, clients’ “perception of Outcome” was used as a dependent variable, predicted by the four domains (as measured variables) of perception of care. This predictive model had an explained variance of 59%.

Future research needs to examine the relationship of perceptions of Outcome and actual client outcomes by conducting provider level analysis of TPS data. The TPS data analysis yielded statistically significant findings for criterion validity. This could be further used to explore the correlation between TPS survey item(s) and domains, with additional client processes and outcomes such as treatment retention, treatment engagement and successful treatment progress at time of discharge. Since no client identifiers were collected as part of the TPS, this type of analysis will need to be conducted at the provider or the county level and linked to individual-level outcomes using multi-level analysis.

Finally, preliminary analysis conducted using TPS data from clients receiving specialty MH services yielded Cronbach’s alphas that were consistent with TPS data from clients receiving specialty SUD services, and demonstrated good reliability (greater than 0.70.) These results indicate the TPS, with slight modifications in item wording, could potentially be used in MH settings with relatively little or no loss of statistical reliability as compared to longer surveys measuring similar domains.

### Limitations and strengths

The present study had a few limitations. First, only clients who presented in person to receive treatment services during a specific five-day survey period were surveyed, and the survey was strictly voluntary. As a result, the sample may have been biased towards clients who were actively engaged in treatment and may have had more favorable perceptions than those who left treatment because of unfavorable perceptions of their treatment experiences. Clients who dropped out of treatment could not be surveyed with the current methodology. Second, the survey administration was limited to the 19 counties that had implemented the DMC-ODS Demonstration Waiver as of August 2018. As a result, the survey data used in the present study may not be representative of all the other California counties that have yet to implement the DMC-ODS Waiver nor of the same counties in the future with more experience in developing their DMC-ODS. Finally, although the TPS questionnaire is standardized and survey administration guidelines were provided, specific procedures to collect survey data varied between providers (e.g., survey questionnaires handed out in groups rather than to each client individually).

Notwithstanding the above limitations, the present study also had some strengths. Clients in all treatment modalities (outpatient/intensive outpatient, residential, OTP/NTP, detox/withdrawal management, and partial hospitalization), 18 years old or older, residing in urban and rural areas and in counties located in different regions of the state (e.g., northern, central, and southern) and with varying population sizes were included in the TPS. This increases the generalizability of the findings to a variety of SUD treatment settings and populations.

## Implications for Behavioral Health

The client TPS was developed to fill a gap in the SUD treatment field for a standardized questionnaire designed to collect actionable data addressing domains that are meaningful to clients and providers in publicly funded SUD treatment delivery systems, while minimizing the burden on clients and county and provider personnel. The study’s findings that the TPS is statistically reliable and a valid tool are important because the survey was designed to be used statewide for program evaluation, external quality review, and local quality improvement purposes under the DMC-ODS Waiver. The research findings should facilitate efforts to conduct statistical analysis of factors related to clients’ perceptions in terms of Access to Care, Quality of Care, Care Coordination, and General Satisfaction that may be associated with perceptions of or actual Outcomes (e.g., length of stay in treatment, substance use).

Furthermore, preliminary results showed the TPS domains were internally consistent when clients receiving MH services were surveyed with a slightly modified questionnaire. This suggests it would be worthwhile to conduct a study with a larger sample of clients receiving MH services that could then include factor analyses and a path analysis to determine the validity and appropriateness of a modified questionnaire for use with client populations receiving MH services.

## Footnotes

1 The federal Medicaid program in California is called “Medi-Cal.” Drug Medi-Cal is a carved-out funding source for substance use disorder services delivered in specialty care settings to Medi-Cal beneficiaries.

2 The link to the MHSIP Consumer Survey on the California Institute for Behavioral Health Solutions website is https://www.cibhs.org/consumer-perception-surveys.

3 The link to the Treatment Perceptions Survey (TPS) website is http://uclaisap.org/dmc-ods-eval/html/client-treatment-perceptions-survey.html.

4 The 19 counties that participated in the 2018 TPS were as follows: Alameda, Contra Costa, Imperial, Los Angeles, Marin, Monterey, Napa, Nevada, Orange, Riverside, San Bernardino, San Diego, San Francisco, San Joaquin, San Luis Obispo, San Mateo, Santa Clara, Santa Cruz, and Yolo.

5 Monterey County changed one of the Care Coordination items from “staff here work with my mental health care providers to support my wellness” to “staff here work with my other community agencies to support my wellness.” In addition, one of the General Satisfaction items used was from an earlier version of the TPS (“I like the services offered here.”), which was subsequently replaced with, “Overall, I am satisfied with the services I received.”.

6 The response rate was calculated as the number of surveys received divided by the number of clients that received services during the survey period as reflected in the administrative DMC claims database. If programs only collected TPS questionnaires from Medi-Cal beneficiaries, the response rate would be 60.9%. If every program sought to collect surveys from all clients, however, then the numerator would be inflated relative to the denominator, since only 67.4% of clients were Medi-Cal beneficiaries in 2018 according to CalOMS-Tx. To adjust for this, the reported response rate would be 67.4% × 60.9% = 41.0%.
